# Pulse contour cardiac output monitoring in acute heart failure patients: Assessment of hemodynamic measurements

**DOI:** 10.1007/s00508-016-1048-z

**Published:** 2016-08-15

**Authors:** Bernhard Wernly, Michael Lichtenauer, Marcus Franz, Michael Fritzenwanger, Bjoern Kabisch, Hans-Reiner Figulla, Christian Jung

**Affiliations:** 1 Clinic of Internal Medicine I, Friedrich-Schiller-University, Jena, Germany; 2 University Clinic of Internal Medicine II, Paracelsus Medical University, Salzburg, Austria; 3 Clinic of Cardiology, Pneumology and Angiology, University Clinic Duesseldorf, Moorenstr. 5, 40225 Duesseldorf, Germany

**Keywords:** Critically ill, Intensive care, PiCCO, Heart failure, Cardiac index

## Abstract

**Background:**

Heart failure is known to be a major public health problem. Fluid redistribution contributes to acute heart failure; therefore, knowledge of hemodynamic parameters could be important for optimizing outcomes. The pulse contour cardiac output monitor PiCCO uses the single thermal indicator technique and pulse contour analysis to calculate hemodynamic parameters of preload, afterload, cardiac output, systemic vascular resistance and extravascular lung water.

**Objectives:**

We primarily aimed to describe values and parameters seen in acute heart failure patients admitted to the intensive care unit (ICU) and secondly to investigate associations between hemodynamic measurements and survival data.

**Material and methods:**

In this study 420 consecutive patients admitted to a tertiary medical university hospital ICU between January 2004 and December 2009 were retrospectively investigated. The study sample was divided into two subgroups: patients monitored by PiCCO (*n* = 47) and those not monitored by thermodilution measurements (*n* = 373). No predetermined treatment algorithm based on knowledge obtained by the PiCCO monitor was used and measurements were individually interpreted by the treating physician. The PiCCO monitor measurements were carried out according to manufacturer’s directions.

**Results:**

Patients with PiCCO monitoring were clinically in poorer health with a mean simplified acute physiology score II (SAPS2) of 45 ± 17 vs. 56 ± 20 (*p* < 0.01). The ICU mortality (22 % vs. 38 %, *p* = 0.02) and, at least as a tendency, long-term-mortality were increased in patients monitored by PiCCO (RR 1.49, 95 % CI 0.96–2.31, *p* = 0.08). We provide hemodynamic measurements in acute heart failure patients: cardiac index (2.7 ± 1.2 l/min/m²) was reduced, preload and extravascular lung water index (EVLWI, 11.5 ± 5.1 ml/kg body weight), representing lung edema, were increased.

**Conclusion:**

We provide real world values for PiCCO parameters in acutely decompensated heart failure. In our study patients who were clinically in poorer health were monitored with PiCCO, resulting in increased mortality in this group. Further prospective studies to investigate the effects of treatment decisions triggered by information obtained by PiCCO monitoring for patients in acute heart failure are needed.

## Introduction

Heart failure is known to be a major public health problem [1, 2]. Congestion manifested as edema, dyspnea and fatigue is the primary reason for acute heart failure hospitalization [3]. Many patients show symptoms of congestion without gaining weight and it is therefore increasingly recognized that fluid redistribution contributes to acute heart failure, for instance via autonomic mechanisms, inflammation and abnormalities in adenosine signalling [4]. Hemodynamic parameters, such as the extravascular lung water index (EVLWI) have been shown to be associated with mortality in acute lung injury/acute respiratory distress syndrome (ALI/ARDS) and after resuscitation [5, 6]. Knowledge of hemodynamic parameters could be of importance because it leads to a more individualized therapy. As both baseline and residual congestion at discharge are associated with increased mortality, sufficient recompensation is a major goal of acute heart failure management [7–9]. High-dose intravenous (i.v.) therapy with diuretics with the addition of vasodilators remains an evidence-based initial approach but there are many other options, such as ultrafiltration, vasopressin antagonists, mineralocorticoid receptor antagonists, inotropic agents and newer substances, such as serelaxin and gut sequestrants [10]. Pulmonary artery catheters have long been the gold standard for cardiac output measurement but concerns regarding safety and cost led to the development of alternative methods, such as the thermodilution method [11, 12]. The pulse contour cardiac output monitor PiCCO (Manufacturer, Town, Country or US State abbreviation) uses the single thermal indicator technique to calculate volumetric parameters and has gained acceptance in many ICUs [13]. It has been shown that both intermittent and continuous cardiac output measurements by PiCCO exhibit good agreement with the gold standard pulmonary artery catheter [14]. The Stewart-Hamilton method is used to calculate cardiac output. The global end-diastolic volume index and intrathoracic blood volume, as robust indicators of cardiac preload and EVLWI, a well-established indicator of lung edema, are calculated using the transpulmonary thermodilution technique [15, 16]. Arterial pulse contour analysis provides parameters of left ventricular function and afterload, such as continuous cardiac output, cardiac index and stroke volume variation (SVV). We primarily aimed to describe values and parameters in acute heart failure patients and secondly to investigate associations of hemodynamic measurements and survival data.

## Methods

### Study subjects

A total of 420 consecutive patients admitted to our tertiary medical University Hospital ICU in Jena between January 2004 and December 2009 were included in this retrospective registry. The study sample was divided into two subgroups: patients monitored by PiCCO (*n* = 47) and those not monitored by thermodilution measurements (*n* = 373). No treatment algorithm based on knowledge obtained by the PiCCO monitor was applied. PiCCO monitor measurements were done according to manufacturer’s instructions. PiCCO monitoring was applied to patients solely according to the decision of the treating physician based on individual clinical experience, clinical examination of the patients and available laboratory values. There were no prefixed patient characteristics by which patients were chosen for undergoing PiCCO. A central venous catheter and an arterial catheter were placed to obtain measurements. Follow-up of patients was performed between May 2013 and November 2013. The primary endpoint of the study was death from any cause. Data on mortality were collected by reviewing medical records or telephone interviews. The study was approved by the local ethics committee of the Medical Faculty of the Friedrich Schiller University of Jena.

### Laboratory analyses

Blood samples were drawn with standard precautions. Laboratory parameters were tested at the Department of Clinical Chemistry at the University Hospital of Jena (Friedrich Schiller University). Some laboratory values were measured repeatedly on the day of admission and we report the maximum or minimum value depending on the clinical relevance which is clearly stated in the article.

### Calculation of SAPS2 and APACHE score

The initial simplified acute physiology score II (SAPS2) and acute physiology and chronic health evaluation (APACHE) scores were calculated by the treating physician within 24 h after admission as previously reported [17, 18].

### Statistical analysis

Statistical analysis was performed using SPSS (IBM Corp. Released 2013. IBM SPSS Statistics for Windows, Version 22.0. Armonk, NY: IBM Corp.). Normally distributed data are given as mean ± standard deviation and compared by Student’s t‑test. Non-normally distributed data are given as median ± interquartile range and compared by the Mann-Whitney U‑test. The χ^2^-test was applied to calculate differences between groups. Cox regression analysis was used to compare and a Kaplan-Meier curve was used to depict survival data.

## Results

The characteristics and parameters measured in the 420 patients are summarized in Table 1 and 47 patients received PiCCO monitoring. The median age of patients receiving PiCCO was significantly lower than those not monitored by PiCCO (69 ± 15 years vs. 73 ± 18 years, *p* < 0.001). Patients receiving PiCCO monitoring represented a clinically more ill patient collective as represented by higher SAPS2 scores (45 ± 17 vs. 56 ± 20, *p* < 0.01) and a tendency towards higher APACHE scores (23 ± 8 vs. 26 ± 9, *p* = 0.09). Furthermore, maximum concentrations on admission of both glucose (9.9 ± 3.4 mmol/l vs. 12.1 ± 5.7 mmol/l, *p* = 0.01) and lactate (3.1 ± 3.8 mmol/l vs. 4.7 ± 3.8 mmol/l, *p* = 0.01), as well as maximum heart frequency (103 ± 23 bpm vs. 116 ± 26 bpm, *p* < 0.001) on admission were significantly higher in patients monitored by PiCCO also reflecting a patient collective with poorer health.

**Table 1:** Characteristics and parameters measured in the 420 patients included in the study are reported here

Factor	No PiCCO	PiCCO	*p* =	
*n*	373	47	–	–
(Minimum) pO2 (kPa)	11.6 ± 3	12.3 ± 3	0.41	–
(Maximum) pCO2 (kPa)	5.6 ± 1	5.3 ± 1	0.94	–
(Minimum) albumin (g/dl)	24 ± 6	21 ± 7	0.03	^*^
(Maximum) base excess (mEq/L)	5.3 ± 5.8	4.9 ± 6.1	0.65	–
(Minimum) hemoglobin (mmol/l)	6.4 ± 1.4	6.1 ± 1.3	0.25	–
(Mean) thrombocytes (×10^9^/l)	193 ± 106	172 ± 113	0.22	–
(Maximum) leucocytes (g/l)	12.7 ± 11.4	13.1 ± 6.4	0.80	–
APACHE score	23 ± 8	26 ± 9	0.09	–
SAPS2 score	45 ± 17	56 ± 20	0.002	^*^
(Maximum) lactate (mmol/l)	3.1 ± 3.8	4.7 ± 3.8	0.01	^*^
(Maximum) glucose (mmol/l)	9.9 ± 3.4	12.1 ± 5.7	0.01	^*^
(Maximum) heart frequency (bpm)	103 ± 23	116 ± 26	<0.001	^*^
Age (years)	72.5 ± 17.7	68.7 ± 15.2	<0.001	^*^

*APACHE* acute physiology and chronic health evaluation, *SAPS2* simplified acute physiology score II, *pO2 partial pressure of O2, pCO2 partial pressure of CO2*, * indicates p<0.05

Table 2 summarizes the hemodynamic measurements in patients admitted to the ICU because of acute heart failure. The mean cardiac index (CI) was 2.7 ± 1.2 l/min/m² representing impaired cardiac function. Both the global end-diastolic volume index (GEDVI, 867.1 ± 207.0 ml/m^2^) and intrathoracic blood volume index (ITBVI, 1033.4 ± 255.8 ml/m^2^) were increased reflecting increased preload. The extravascular lung water index (EVLWI, 11.5 ± 5.1 ml/kg body weight) was increased, representing lung edema. Afterload was low as expected with a systemic vascular resistance index (SVRI) of 942 ± 473.0 dyn × sec × cm^−5^ × m^2^.

**Table 2:** Hemodynamic measurements of patients admitted to ICU because of acute heart failure obtained by PiCCO

Hemodynamic measurement	Unit	Mean	SD	*n* =	Normal range
Cardiac index (CI)	(l/min/m^2^)	2.7	±1.2	47	3–5
Intrathoracic blood volume (ITBVI)	(ml/m^2^)	1033.4	±255.8	21	850–1000
Systemic vascular resistance (SVR)	(dyn × sec × cm^−5^ × m^2^)	942.7	±473.0	41	1700–2400
Global end-diastolic volume (GEDV)	(ml/m^2^)	867.1	±207.0	18	680–800
Extravascular lung water (EVLW)	(ml/kg body weight)	11.5	±5.1	25	3–7
Cardiac output (CO)	(l/min)	5.3	±2.4	47	4.5–5
Central venous pressure (CVP)	mmHg	14.7	±6.7	37	0–9

**Fig. 1:**
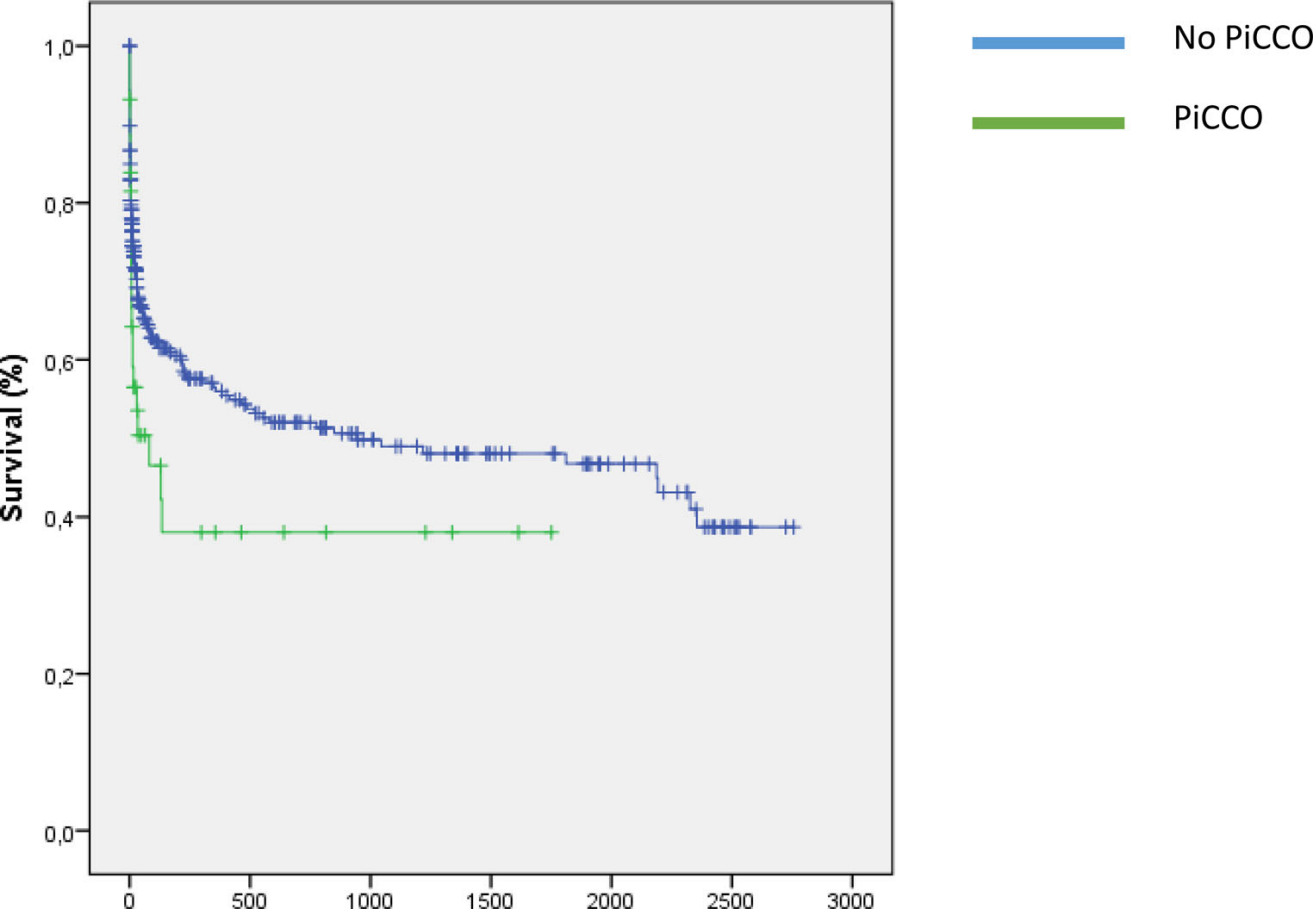
In our study sample of acute heart failure patients which was retrospectively divided into two subgroups receiving PiCCO (green line) and not receiving PiCCO (blue line), long-term mortality was higher in those monitored by PiCCO (RR 1.49, 95 % CI 0.96–2.31, *p* = 0.08)

The ICU survival (RR = 1.8, OR = 2.3, *p* = 0.02, 18 patients out of 47 monitored by PiCCO died compared to 80 patients out of 373 without PiCCO monitoring) was worse in patients receiving PiCCO monitoring. Long-term mortality was at least a tendency to be increased in patients monitored by PiCCO (RR 1.49, 95 % CI 0.96–2.31, *p* = 0.08) (Fig. 1).

Furthermore, hemodynamic measurements were investigated for potential use of risk stratification and prediction of survival; therefore, we divided patients into two groups above and below the median of our study population regarding hemodynamic measurements of acute heart failure patients admitted to the ICU. There were no differences regarding both ICU mortality, e. g. when stratified for EVLWI (50 % vs. 38.46 %, not significant, 6 out of 12 patients with an EVLWI above the median died as compared to 5 out of 13 patients below the median) and cardiac output (39.13 % vs. 37.5 %, not significant, 9 out of 23 patients with EVLWI above the median died as compared to 9 out of 24 patients below the median).

## Discussion

We describe hemodynamic parameters and measurements in patients admitted to the ICU because of acute heart failure. This could be important for further studies to evaluate effects on mortality of PiCCO monitoring in acute heart failure patients. Besides high-dose i. v. diuretics there are a variety of other therapeutic options available for patients with acute heart failure. Several parameters of preload and afterload, cardiac output, SVR and EVLW can be measured and, within limits, controlled by the physician. It could be possible to develop therapeutic algorithms for acute heart failure to guide or at least optimize therapy by measurements obtained by PiCCO as introduced for subarachnoid hemorrhage, sepsis and acute respiratory distress syndrome [19].

In our study acute heart failure patients receiving PiCCO monitoring had increased ICU mortality and at least a tendency towards increased long-term mortality. There were no serious adverse events regarding the thermodilution measurements reported but patients receiving PiCCO were clinically in poorer health, as SAPS2 scores and maximum concentrations of lactate and glucose on the day of admission were significantly higher in these patients. Lactate is known to be an indicator of tissue hypoperfusion and adverse outcome [20]. Increased glucose concentrations are also known to be associated with worse survival in patients suffering from cardiovascular disease [21]. Furthermore, patients receiving PiCCO had a higher maximum heart frequency on the day of admission, which is known to be associated with increased mortality [22, 23]. Most probably this excess mortality is because clinically more ill patients, who have a higher risk of death, are more likely to receive PiCCO monitoring. Interestingly, in our study the median age of patients receiving PiCCO monitoring was lower than those without PiCCO measurements. As there were no prefixed selection criteria for which patients should receive PiCCO this is most probably due to selection bias: Physicians were more likely to use PiCCO, a minor but still invasive procedure, in patients clinically ill and of a relatively young age.

Risk stratification based on hemodynamic measurements obtained by PiCCO failed. There were no differences regarding mortality when we split our patients into subcohorts above and below the median values of, e. g. EVLWI and cardiac output. As already discussed, in this study mainly patients who were clinically ill received PiCCO: therefore, we think that the irrelevance of hemodynamic measurements for prediction of mortality is at least partly due to selection bias and we speculate on an association of mortality with those values in a more inhomogeneous patient collective.

Future prospective studies are needed to determine (i) algorithms which acute heart failure patients could benefit from and therefore should receive PiCCO and (ii) how to optimally interpret hemodynamic measurements obtained by PiCCO monitoring in those patients to optimize outcome. It is therefore necessary to develop treatment algorithms based on the hemodynamic measurements reported here and by Ritter et al. and test them in prospective clinical trials [24].

## Limitations

Our study has several limitations: it is of retrospective design and single-centered. Furthermore, as PiCCO measurements were applied to patients according to the treating physician’s decision, there was certainly a selection bias as we speculate that clinically ill patients, being more tachypneic, cyanotic and showing signs of hypoperfusion, are more likely to receive more invasive treatment. For an analysis of mortality our study cohort of 47 patients undergoing PiCCO monitoring is certainly underpowered.

## Conclusion

In this study we report important real life hemodynamic values obtained by thermodilution measurements and pulse contour analysis using the PiCCO monitor in 47 patients admitted to the ICU for acute heart failure. Patients monitored by PiCCO were clinically in poorer health and showed increased ICU mortality, most probably due to selection bias. To evaluate a possible benefit of PiCCO measurement on survival of acute heart failure patients, prospective studies with prefixed criteria for which patients should receive PiCCO are needed. Furthermore, prefixed algorithms for therapeutic decisions based on measurements obtained by PiCCO need to be tested in prospective studies against the standard of care. For these future algorithms we provide important real world values of hemodynamic parameters in a severely ill cohort of acute heart failure patients.
